# Sulcal Hyperintensity as an Early Imaging Finding in Cerebral Amyloid Angiopathy–Related Inflammation

**DOI:** 10.1212/WNL.0000000000210084

**Published:** 2024-11-25

**Authors:** Larysa Panteleienko, Gargi Banerjee, Dermot Henry Mallon, Victoria Harvey, Rupert Oliver, Gary Hotton, William Knight, Sayan Datta, Michael S. Zandi, Hans Rolf Jäger, David John Werring

**Affiliations:** From the Stroke Research Centre (L.P., D.J.W.), Department of Brain Repair and Rehabilitation, UCL Queen Square Institute of Neurology, London, United Kingdom; Department of Neurology (L.P.), Bogomolets National Medical University, Kyiv, Ukraine; MRC Prion Unit at UCL (G.B.), Institute of Prion Diseases; National Hospital for Neurology and Neurosurgery (G.B., R.O., G.H., H.R.J.), Queen Square, University College London Hospitals NHS Foundation Trust; Neuroradiological Academic Unit (D.H.M., H.R.J.), UCL Queen Square Institute of Neurology; Lysholm Department of Neuroradiology (D.H.M.), National Hospital for Neurology and Neurosurgery; National Hospital for Neurology and Neurosurgery (V.H.), Queen Square, University College London Hospitals NHS Foundation Trust; Torbay and South Devon NHS Foundation Trust (W.K.); York and Scarborough Teaching Hospitals NHS Foundation Trust (S.D.); Queen Square Institute of Neurology and National Hospital for Neurology and Neurosurgery (M.S.Z.), University College London Hospitals NHS Foundation Trust; and Department of Neuroinflammation (M.S.Z.), UCL Queen Square Institute of Neurology, London, United Kingdom.

## Abstract

**Background and Objectives:**

Cerebral amyloid angiopathy–related inflammation (CAA-ri) is a subtype of CAA with distinct clinical and radiologic features. Existing diagnostic criteria require the presence of characteristic asymmetrical white matter hyperintensity (WMH), together with classical hemorrhagic neuroimaging markers of CAA. There are limited data for other diagnostic neuroimaging markers of CAA-ri.

**Methods:**

This is a case series from a specialist hospital intracerebral hemorrhage service.

**Results:**

We describe 4 patients with CAA-ri who had regions of sulcal hyperintensity, with or without gyral swelling at clinical presentation, but did not fulfill current diagnostic criteria because of the absence of typical asymmetric WMH on brain MRI. All 4 patients were subsequently diagnosed with CAA-ri; three later developed asymmetric WMHs with disease relapse, and 2 had pathologically proven CAA-ri; 1 patient had both.

**Discussion:**

Regions of sulcal hyperintensity, sometimes with associated gyral swelling, can be an early imaging finding in CAA-ri. These neuroimaging markers could potentially improve the accuracy of existing diagnostic criteria for CAA-ri to allow earlier diagnosis and treatment without biopsy in patients with atypical presentations.

## Introduction

Current clinical-radiologic diagnostic criteria for cerebral amyloid angiopathy–related inflammation (CAA-ri)^1^ require the presence of asymmetrical white matter hyperintensities (WMH) and hemorrhagic neuroimaging markers of CAA (cerebral microbleeds [CMBs], cortical superficial siderosis [cSS], or both). Although these criteria can allow noninvasive diagnosis, reports of patients with proven CAA-ri who do not fulfill them highlight the need for improved diagnostic accuracy.^2-4^ Other neuroimaging features associated with CAA-ri include leptomeningeal enhancement, regions of sulcal hyperintensity, small scattered diffusion-weighted imaging (DWI) hyperintense lesions, and gyral swelling. We describe 4 patients who initially presented without asymmetric WMH, but were subsequently diagnosed with CAA-ri (3 developed asymmetric WMH and 2 had pathologically confirmed CAA-ri [1 patient had both]).

## Case Reports

Clinical and investigation-related findings for all cases are summarized in the Table.

**Table:** Summary of Main Clinical and Radiologic Characteristics

	Case 1	Case 2	Case 3	Case 4
Age	77	66	53	70
Sex	F	M	M	F
Presenting symptoms at onset	Subacute confusion, agitation disorientation	Subacute confusion, disorientation	Headache, TFNEs, memory decline	Severe migraine-like headache
Relapse	Yes, after 5 mo	No	Yes, after 3 and then 24 mo	Yes, after 10 mo
Biopsy-proven CAA-ri	+	+	—	—
MRI features at presentation				
Asymmetric cortical-subcortical WMH	—	—	—	—
Sulcal hyperintensity	+	+	+	+
Gyral swelling	—	—	+	—
Leptomeningeal enhancement	+	+	—	Unknown
Small scattered DWI	—	+	—	—
MRI features at relapse				
Asymmetric cortical-subcortical WMH	+	Not applicable (no relapse)	+	+
Sulcal hyperintensity	+	Not applicable (no relapse)	—	+
Gyral swelling	+	Not applicable (no relapse)	—	—
Leptomeningeal enhancement	+	Not applicable (no relapse)	+	+
Small scattered DWI	+	Not applicable (no relapse)	—	—

Abbreviations: DWI = diffusion-weighted imaging; SWI = susceptibility-weighted imaging; TFNE = transient focal neurologic episode; WMH = white matter hyperintensity.

### Case 1

A 77-year-old woman was admitted with a 3-week history of confusion, disorientation, agitation, and word-finding difficulties. Brain MRI showed extensive regions of sulcal hyperintensity (SH) (Figure, A) and leptomeningeal enhancement (Figure, B) over both hemispheres; no blood-sensitive sequences were obtained. Blood tests for HIV, hepatitis B and C, syphilis, and Lyme were negative. CSF showed 1 × 10^6^/L lymphocytes, elevated protein of 2.63 g/L, negative viral PCR, microscopy, and bacterial culture. IV methylprednisolone (1 g/d for 5 days) was administered, with marked clinical and radiologic improvement. Five months later, the patient again became confused, disorientated, and developed visual hallucinations. MRI showed asymmetrical confluent WMH (Figure, C), SH, and leptomeningeal enhancement over the left parieto-occipital area, multiple small subcortical DWI lesions, and 2 lobar CMBs on susceptibility-weighted images (SWIs). Brain biopsy confirmed widespread amyloid beta (Aβ) deposition in leptomeningeal and cortical blood vessels and mild-to-moderate lymphohistiocytic perivascular infiltrates (extending into the vessel wall in places) with no giant cells. CAA-ri relapse was considered likely, and the patient had a further course of IV methylprednisolone (1 g/d for 5 days) with oral prednisolone (60 mg daily) thereafter. Although there was radiologic improvement, disorientation, agitation, and visual hallucinations persisted. MRI 2 months later showed improvement of the WMH and leptomeningeal enhancement, but with several new small cortical and subcortical DWI lesions (Figure, D) and multiple microhemorrhages. The patient received further immunosuppressive therapy with mycophenolate mofetil (2,000 mg daily) and 2 infusions of rituximab. Four months later, hallucinations had resolved, but she had developed dementia and required constant support in her activities of daily living. MRI showed near-complete resolution of the WMH and leptomeningeal enhancement, with no new DWI lesions, but with marked subcortical volume loss.

**Figure 1:**
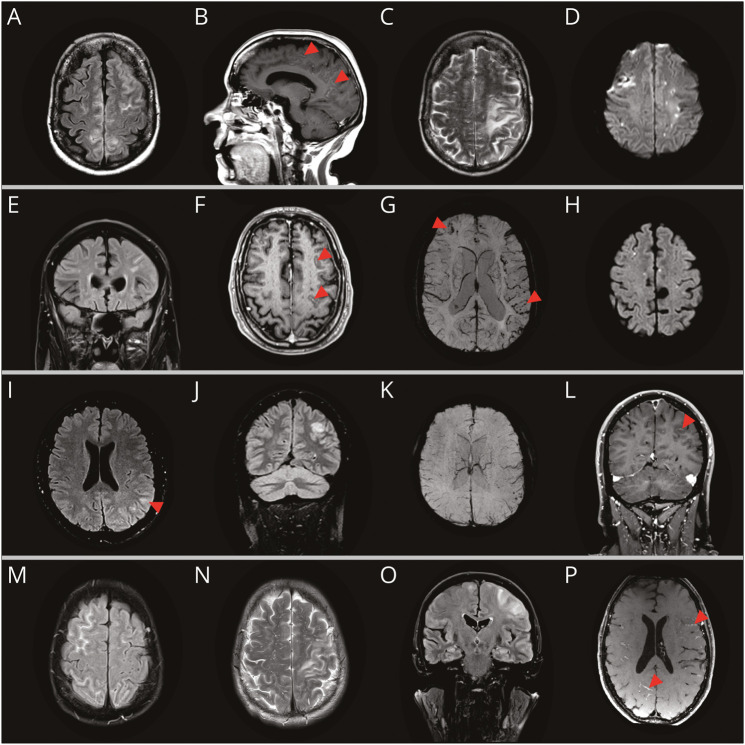
Representative MRI Findings for the Cases Described Case 1 (A–D): MRI at initial presentation showed sulcal hyperintensity (SH) on fluid attenuated inversion recovery (FLAIR) images (A), leptomeningeal contrast enhancement (B, red arrowheads); MRI at relapse 5 months after initial presentation showed asymmetric white matter hyperintensity (WMH), over the left parietal and occipital lobes (C), in addition to multiple cortical and subcortical foci of restricted diffusion on DWI sequences (D). Case 2 (E–H): MRI at presentation showed SH over the left frontal lobe on FLAIR images (E), leptomeningeal contrast enhancement (F, red arrowheads), lobar cerebral microbleeds (CMB, red arrowheads), and areas of cortical superficial siderosis (cSS), mostly over the left frontal lobe on SWI (G), and multiple subcortical DWI lesions (H). Case 3 (I–L): MRI at initial presentation showed a subtle area of cortical swelling and SH in the left parietal region (I, red arrowhead); MRI at relapse showed a new patch of the WMH in the left temporoparietal region (J), associated with a cluster of CMB (K). The follow-up image 2 years later showed leptomeningeal enhancement in the left temporoparietal region (L, red arrowhead). Case 4 (M–P): MRI at initial presentation showed SH with gyral swelling on the FLAIR image over the right frontal lobe and occipital lobes bilaterally (M); MRI at relapse 9 months later showed areas of WMH with mild parenchymal swelling on T2 (N) and FLAIR (O) images primarily affecting the left perirolandic region, and leptomeningeal contrast enhancement (P, red arrowheads).

### Case 2

A 66-year-old man was admitted with 2 weeks of confusion, behavioral changes, and word-finding difficulties. Brain MRI on admission showed left frontal SH (Figure, E), left frontal and temporal leptomeningeal contrast enhancement (Figure, F), multiple lobar CMBs, and areas of focal cSS on SWIs (Figure, G). There were several small subcortical DWI lesions (Figure, H). CSF showed 10 × 10^6^/L lymphocytes, elevated protein (1.41g/L) with negative oligoclonal bands. CSF viral PCR, microscopy, bacterial culture, and anti-neuronal antibodies were negative. IV methylprednisolone (1g/day for 5 days) was administered, with some clinical improvement. Brain MRI 1 month later showed resolution of the SH and no further progression of cSS or CMB. However, there were multiple new scattered subcortical DWI lesions (not shown). Brain biopsy showed thickened leptomeningeal and cortical vessels with Aβ deposition; some were cuffed with CD3+ T cells and CD68+ macrophages. Diffuse cortical Aβ-positive plaques without tau pathology were noted. The patient continued tapering oral prednisolone with the addition of mycophenolate mofetil 2,000 mg daily, with marked clinical improvement. Two months later, the patient had a generalized tonic-clonic seizure but recovered with no new MRI changes. One year after initial presentation, the patient had developed significant cognitive decline with pronounced anterior dysfunction, but no further seizures.

### Case 3

A 53-year-old man presented after developing daily intermittent tingling involving the right side of his face, lasting a couple of minutes and occurring twice or thrice a week. The frequency increased, occurring up to twice a day over approximately 3 months. During this time, the patient also experienced short-term memory problems, and intermittent daily headaches without migranous features. Brain MRI at presentation showed subtle cortical swelling and SH in the left parietal region (Figure, I); blood-sensitive sequences were not obtained. MRI 3 months later showed new confluent WMH in the left anterior temporal lobe (Figure, J) associated with a cluster of CMBs (Figure, K). Immunosuppression was not prescribed. MRI 2 years after original presentation showed resolution of the left anterior temporal WMH, but a new area of the WMH in the left temporoparietal region (not shown) with new overlying leptomeningeal enhancement (Figure, L).

### Case 4

A 70-year-old woman presented with a 4-day history of severe, episodic, bilateral periorbital headaches preceded by bilateral visual blurring and “flashing lights” lasting for 1 hour; she had 3 such episodes. Brain MRI showed right frontal and bilateral occipital regions of SH with associated gyral swelling (Figure, M). Blood-sensitive sequences were not obtained. MRI at 5 months showed resolution of the SH and evidence of several lobar CMBs and focal cSS. Nine months later, she had a mild head injury with a brief loss of consciousness. Six weeks later, she experienced multiple transient focal neurologic episodes (TFNEs) (approximately 40 over 4 weeks) of numbness and tingling in her right arm, which smoothly and slowly moved up into the face and tongue over 10 to 20 minutes. These subsequently occasionally involved the left side of the body. Brain MRI showed asymmetrical WMH associated with mild parenchymal swelling on T2 and fluid attenuated inversion recovery (FLAIR) images, primarily affecting the left perirolandic region and right frontal lobe (Figure, N and O), the latter associated with leptomeningeal contrast enhancement (Figure, P). CSF analysis showed 2 × 10^6^/L lymphocytes; elevated protein (1.18 g/L); but negative viral, microbial, and autoimmune tests. She was treated with IV methylprednisolone (1 g/d for 5 days), followed by oral prednisolone (60 mg daily), with near-complete resolution of TFNEs. Brain MRI 3 weeks after discharge showed resolution of WMH and sulcal hyperintensities and no leptomeningeal enhancement.

## Discussion

We describe 4 patients with a final diagnosis of CAA-ri who did not have asymmetric WMH at presentation, which current data suggest is the primary feature for fulfilling the existing diagnostic criteria.^5^ Sulcal hyperintensities were present in all cases, with associated gyral swelling in 1 patient. All patients subsequently fulfilled existing CAA-ri criteria (3 later developed characteristic asymmetric WMH and 2 had biopsy-proven CAA-ri). Our findings suggest that regions of SH, gyral swelling, or both can be early imaging findings in CAA-ri in patients who do not fulfill current diagnostic criteria; these neuroimaging markers expand the spectrum of CAA-ri and could potentially allow earlier diagnosis and treatment without biopsy in patients with atypical presentations.

The key neuroimaging biomarker differentiating CAA-ri from noninflammatory CAA is asymmetric WMH, considered to be due to vasogenic edema.^1^ However, recent case reports have described additional radiologic features in CAA-ri, including multiple DWI hyperintense lesions, leptomeningeal enhancement, gyral swelling, and sulcal hyperintensities^4,6^; the latter 2 markers are also seen in ARIA (amyloid-related imaging abnormalities), which occur after treatment with anti-Aβ immunotherapy for Alzheimer disease, which, like CAA-ri, is hypothesized to be an immune-mediated attempt to clear parenchymal Aβ.^7,8^

SWI MRI sequences were not initially obtained in 3 of the 4 patients described, significantly delaying diagnosis. This emphasizes the need for blood-sensitive MRI sequences (with SWI preferred over T2*-weighted images because of higher sensitivity) in all patients with suspected CAA-ri.

Establishing a diagnosis of CAA-ri in patients with a characteristic clinical presentation but without asymmetric WMH at presentation currently requires brain biopsy according to available diagnostic criteria. Additional neuroimaging features (regions of SH, with or without gyral swelling) might allow for earlier diagnosis and treatment of CAA-ri without brain biopsy in such patients; further research is needed to determine whether they can improve the diagnostic accuracy of current diagnostic criteria for CAA-ri.

## Appendix Authors

**Table:**

Name	Location	Contribution
Larysa Panteleienko, MD, PhD	Stroke Research Centre, Department of Brain Repair and Rehabilitation, UCL Queen Square Institute of Neurology, London, United Kingdom; Department of Neurology, Bogomolets National Medical University, Kyiv, Ukraine	Drafting/revision of the manuscript for content, including medical writing for content; major role in the acquisition of data; study concept or design; analysis or interpretation of data
Gargi Banerjee, BMBCh, MRCP, PhD	MRC Prion Unit at UCL, Institute of Prion Diseases, National Hospital for Neurology and Neurosurgery, Queen Square, University College London Hospitals NHS Foundation Trust, London, United Kingdom	Drafting/revision of the manuscript for content, including medical writing for content; study concept or design
Dermot Henry Mallon, BSc, MBChB, MSc, PhD, FRCR	Neuroradiological Academic Unit, UCL Queen Square Institute of Neurology, Lysholm Department of Neuroradiology, National Hospital for Neurology and Neurosurgery, London, United Kingdom	Analysis or interpretation of data
Victoria Harvey, Sc, PGDip	National Hospital for Neurology and Neurosurgery, Queen Square, University College London Hospitals NHS Foundation Trust, United Kingom	Drafting/revision of the manuscript for content, including medical writing for content; additional contributions (in addition to one or more of the above criteria)
Rupert Oliver, MD, PhD	National Hospital for Neurology and Neurosurgery, Queen Square, University College London Hospitals NHS Foundation Trust, United Kingdom	Drafting/revision of the manuscript for content, including medical writing for content; additional contributions (in addition to one or more of the above criteria)
Gary Hotton, MD	National Hospital for Neurology and Neurosurgery, Queen Square, University College London Hospitals NHS Foundation Trust, United Kingdom	Drafting/revision of the manuscript for content, including medical writing for content; additional contributions (in addition to one or more of the above criteria)
William Knight, MD, MRCP	Torbay and South Devon NHS Foundation Trust, United Kingdom	Drafting/revision of the manuscript for content, including medical writing for content; additional contributions (in addition to one or more of the above criteria)
Sayan Datta, MD, MRCP	York and Scarborough Teaching Hospitals NHS Foundation Trust, United Kingdom	Drafting/revision of the manuscript for content, including medical writing for content; additional contributions (in addition to one or more of the above criteria)
Michael S. Zandi, MA, MB, BChir, MA, PhD, MRCP, FRCP	Queen Square Institute of Neurology and National Hospital for Neurology and Neurosurgery, University College London Hospitals NHS Foundation Trust, Department of Neuroinflammation, UCL Queen Square Institute of Neurology, London, United Kingdom	Drafting/revision of the manuscript for content, including medical writing for content; additional contributions (in addition to one or more of the above criteria)
Hans Rolf Jäger, MD	National Hospital for Neurology and Neurosurgery, Queen Square, University College London Hospitals NHS Foundation Trust; Neuroradiological Academic Unit, UCL Queen Square Institute of Neurology, London, United Kingdom	Drafting/revision of the manuscript for content, including medical writing for content
David John Werring, MBBS, BSc, FRCP, PhD	Stroke Research Centre, Department of Brain Repair and Rehabilitation, UCL Queen Square Institute of Neurology, National Hospital for Neurology and Neurosurgery, University College London Hospitals NHS Foundation Trust, United Kingdom	Drafting/revision of the manuscript for content, including medical writing for content; Study concept or design
